# Orthopedic Injury Profiles in Adolescent Elite Athletes: A Retrospective Analysis From a Sports Medicine Department

**DOI:** 10.3389/fphys.2019.00544

**Published:** 2019-05-09

**Authors:** Michael Cassel, Juliane Müller, Othmar Moser, Mares Elaine Strempler, Judith Reso, Frank Mayer

**Affiliations:** ^1^Outpatient Clinic, Sports Medicine and Orthopedics, University of Potsdam, Potsdam, Germany; ^2^Professorship for Physiotherapy, Exercise Science and Applied Biomechanics, Department of Computer Science – Therapy Sciences, Trier University of Applied Sciences, Trier, Germany; ^3^Applied Sport, Technology, Exercise and Medicine Research Centre, College of Engineering, Swansea University, Swansea, United Kingdom; ^4^Division of Endocrinology and Diabetology, Department of Internal Medicine, Medical University of Graz, Graz, Austria

**Keywords:** overuse injuries, epidemiology, complaints, symptoms, risk factors, sports

## Abstract

**Aim:** The aim of the study was to identify common orthopedic sports injury profiles in adolescent elite athletes with respect to age, sex, and anthropometrics.

**Methods:** A retrospective data analysis of 718 orthopedic presentations among 381 adolescent elite athletes from 16 different sports to a sports medical department was performed. Recorded data of history and clinical examination included area, cause and structure of acute and overuse injuries. Injury-events were analyzed in the whole cohort and stratified by age (11–14/15–17 years) and sex. Group differences were tested by chi-squared-tests. Logistic regression analysis was applied examining the influence of factors age, sex, and body mass index (BMI) on the outcome variables area and structure (α = 0.05).

**Results:** Higher proportions of injury-events were reported for females (60%) and athletes of the older age group (66%) than males and younger athletes. The most frequently injured area was the lower extremity (47%) followed by the spine (30.5%) and the upper extremity (12.5%). Acute injuries were mainly located at the lower extremity (74.5%), while overuse injuries were predominantly observed at the lower extremity (41%) as well as the spine (36.5%). Joints (34%), muscles (22%), and tendons (21.5%) were found to be the most often affected structures. The injured structures were different between the age groups (*p* = 0.022), with the older age group presenting three times more frequent with ligament pathology events (5.5%/2%) and less frequent with bony problems (11%/20.5%) than athletes of the younger age group. The injured area differed between the sexes (*p* = 0.005), with males having fewer spine injury-events (25.5%/34%) but more upper extremity injuries (18%/9%) than females. Regression analysis showed statistically significant influence for BMI (*p* = 0.002) and age (*p* = 0.015) on structure, whereas the area was significantly influenced by sex (*p* = 0.005).

**Conclusion:** Events of soft-tissue overuse injuries are the most common reasons resulting in orthopedic presentations of adolescent elite athletes. Mostly, the lower extremity and the spine are affected, while sex and age characteristics on affected area and structure must be considered. Therefore, prevention strategies addressing the injury-event profiles should already be implemented in early adolescence taking age, sex as well as injury entity into account.

## Introduction

Adolescence represents a critical period in life span, characterized by a tremendous pace in growth influencing skeletal biomechanics, muscle strength and thus athletic performance (Caine et al., 2006; Bergeron et al., 2012). In particular, it is a challenging phase for adolescent athletes as their body has to adapt to growth in addition to systematic training processes and high impact forces in their individual sports (Jacobsson et al., 2012, 2013). Especially contact sports (i. e., game sports) tremendously increase the injury risk among adolescent athletes (Emery and Tyreman, 2009).

The musculoskeletal system of the growing athlete shows typical characteristics, including the existing epiphyseal and the apophyseal zones, making it more vulnerable for specific injuries (Caine et al., 2006; Engebretsen et al., 2010; Launay, 2015). Besides epiphyseal injuries, mainly traumatic and overuse injuries of the apophysis are common pathologies (Caine et al., 2006). Tendons are considerably stronger and more elastic than their apophyseal insertion zones, resulting in locations susceptible for injuries and complaints (Blankstein et al., 2001; Cassas and Cassettari-Wayhs, 2006; Engebretsen et al., 2010; Maffulli et al., 2011). Bones are less mineralized and have reduced fracture toughness in young individuals resulting in a higher proportion of fractures concerning all sustained injuries during adolescence (Kontulainen et al., 2007; Darrow et al., 2009). Furthermore, it has been frequently reported that young adolescent athletes have an increased risk of sustaining severe and overuse skeletal injuries compared to non-athletes (Villemure and Stokes, 2009; Stracciolini et al., 2013; Cassel et al., 2018).

The majority of studies analyzing injury profiles in adolescents investigated the general pediatric or adolescent population participating in sports on a recreational level. Fridman et al. (2013) evaluated sports-related injuries in different sports of children and youth 5 to 19 years of age presenting to Canadian emergency departments (Fridman et al., 2013). They reported differences in the injury profiles related to age groups (5–9, 10–14, 15–19 years), sex and type of sports with highest rates in the age group 10–14 years (57%) and males (71%) affected more often than females (Fridman et al., 2013). Stracciolini et al. (2013, 2014) focused their work on pediatric sports injuries (overuse vs. acute/traumatic) in 2133 subjects exercising in ‘organized physical activity’ with respect to sex, age group (5–12, 13–17 years) and type of sports practiced. Results indicated that females as well as the older age group present more often with overuse symptoms

(i.e., patellofemoral pain syndrome) and males as well as younger age group subjects show up more often with traumatic injuries (including fractures, apophysitis or osteochondrosis) (Stracciolini et al., 2013, 2014). In a recent additional analysis Stracciolini et al. (2015a) had “a closer look” at their data concerning isolated overuse injuries within a cohort of 1614 physically active adolescents. Substantial differences in the proportion of overuse injuries were observed between males and females (60% vs. 43%, respectively). Injury location differed significantly by sex with females showing up more frequently for lower extremity, hip/pelvis, and spine injuries, while males sustained more injuries to the head, chest, and upper extremity (Stracciolini et al., 2015a). In contrast, very little is known concerning sports injury and complaint profiles of adolescent elite athletes (Steffen and Engebretsen, 2010).

Differentiation between acute and overuse injuries in adolescent elite athletes is not well established (Roos et al., 2015). In a recently published “International Olympic Committee” consensus statement on youth athletic development, the authors stated that the competitive careers of youth athletes are too often temporarily halted or permanently derailed by overuse injuries (Bergeron et al., 2015). In order to protect adolescents athletes’ health and to allow an optimal development of their athletic performance, it is essential to identify common musculoskeletal injuries in this special cohort (Caine et al., 2008). However, data evaluating frequency and location of different types of over the whole season in adolescent elite athletes is sparse (Caine et al., 2008; Cassel et al., 2015). Establishing the extent of the sports injury problem is the first step in the sequence of injury prevention and is followed by the identification of injury etiology and contributing risk factors (van Mechelen et al., 1992; Timpka et al., 2014). Therefore, the aim of the present study was to identify common locations of musculoskeletal injuries in high performance adolescent elite athletes with stratification to age, sex, and anthropometrics.

## Materials and Methods

### Study Design and Subjects

In 2007 a medical surveillance system for health care of adolescent athletes in the elite schools of sport in the federal state of Brandenburg (Germany) was implemented (Mayer et al., 2012). Elite schools of sports are special types of schools, ensuring that talented young elite athletes are encouraged to develop their full athletic potential while also attaining their educational qualifications (Mueller et al., 2014). Athletes are enabled to train in their specific sport discipline approximately 15–20 h per week within an organized school schedule (Mayer et al., 2012; Cassel et al., 2015). In order to monitor their health status a medical database was implemented. Medical consultations of each athlete due to orthopedic reasons were documented in a local university outpatient clinic, responsible for athletes’ medical care. Data of medical history as well as clinical examination (including diagnosis), evaluated by a sports medicine physician (ten physicians participated after receiving instructions and training for using the database), were digitally stored in a web-based database (ProWebDB, Germany). Besides defining the cause of orthopedic injury, anthropometric data, type of sport and squad membership were registered. In addition, first and follow-up presentations of the same medical complaint were distinguished. Before entrance to the medical surveillance system, parents of each athlete signed written informed consent of participation as well as the agreement for the anonymous scientific analysis and publication of medical data. The study was approved by the local ethics committee (faculty of human science) of the Potsdam University.

The present study used a retrospective analysis design of medical presentations between August 2009 and March 2015 of injury-events among adolescent athletes from an elite school of sport (average of approximately 400 athletes per school year) between 11 and 17 years of age. Only first contacts of an athlete with symptoms were included in this investigation, while data of follow-up examinations for the same injury were excluded. In total 795 presentations of injury-events matched the inclusion criteria. Range check and exclusion of incomplete datasets (i.e., missing anthropometric data or diagnosis) finally resulted in the sum of 718 presentations (m/f: 291/427; age group 11–14/15–17: 243/475) from 381 adolescent elite athletes originating from 16 different sports (Table 1). The anthropometric data of the complete dataset as well as of the subgroups (sex and age) are displayed in Table 2. Furthermore, injuries were classified according to their origin in “acute” (traumatic onset with a specific, identifiable event) or “overuse” (pain or discomfort caused by repeated micro traumata not directly associated with a single, identifiable traumatic event) (Fuller et al., 2006).

**TABLE 1:** Distribution of injury-events for sports (sports categorization*) performed of elite adolescent athletes in the complete dataset (n; %) as well as between the subgroups [age groups and gender, (n)].

Sport	Sports category*	Count (n)	(%)	n (m/f)	n (11–14/15–17)
**Total**		718	100	291/427	243/475
Canoe sprint	E	109	15	46/63	35/74
Cycling	E	5	0.5	4/1	0/5
Gymnastics	S	12	2	12/0	1/11
Handball	BC	55	8	55/0	8/47
Horse riding	E	4	0.5	1/3	0/4
Judo	BC	22	3	12/10	9/13
Modern pentathlon	S	34	5	17/17	17/17
Rowing	E	103	14	40/63	34/69
Soccer	BC	40	6	3/37	14/26
Swimming	E	50	7	27/23	26/24
Track and field	S	180	25	42/138	74/106
Triathlon	E	46	6	17/29	7/39
Volleyball	BC	30	4	1/29	8/22
Water polo	BC	3	0.5	2/1	0/3
Weight lifting	S	17	2	10/7	5/12
Wrestling	BC	8	1.5	2/6	5/3

*BC, ball and combat sports; E, endurance sports; S, explosive strength sports [modified according to Cassel et al. (2017); Müller et al. (2017)].

**TABLE 2:** Anthropometric data, area, structure and cause of all injury-events in the complete dataset (n; %) and within the subgroups [age groups and sex as well as for differentiation of acute vs. overuse injuries; (%; X^2^ *p*-value)].

		Full sample		Age group 11–14	Age group 15–17	Males	Females	Acute injuries	Overuse injuries
Age (y)		15.5 ± 1.5		13.8 ± 0.8	16.4 ± 0.8	15.8 ± 1.5	15.3 ± 1.4	15.6 ± 1.6	15.5 ± 1.5
Height (cm)		172 ± 11		166 ± 10	175 ± 10	178 ± 11	167 ± 8	172 ± 11	172 ± 11
Weight (kg)		63 ± 13		54 ± 10	67 ± 12	69 ± 14	59 ± 11	62 ± 13	63 ± 13
BMI (kg/m^2^)		21.1 ± 2.6		19.5 ± 2.1	21.8 ± 2.5	21.5 ± 2.6	20.7 ± 2.6	21.0 ± 2.5	21.0 ± 2.6

**Presentations (n)**		**718**	**100**	**243**	**475**	**291**	**427**	**140**	**578**
		**(n)**	**(%)**	**(%)**	**(%)**	**(%)**	**(%)**	**(%)**	**(%)**

**Area**									
	Head/Neck	14	2	2	2	2.5	1.5	3.5	1.5
	Upper Ex	90	12.5	13	12	18	9	11.5	13
	Core/Thorax	53	7.5	6	8	6	8	5	8
	Lower Ex	340	47	48	47	47.5	47	73.5	41
	Spine	219	30.5	31	30	25.5	34	5	36.5
	Other	2	0.5	0	1	0.5	0.5	1.5	0
	X^2^ (*p*-value)			0.834		0.005		<0.001	
**Structure**									
	Ligament	32	4.5	2	5.5	5	4	21	0.5
	Joint	243	34	30.5	36	35	33	26.5	35.5
	Skin	10	1.5	1.5	1	1.5	1.5	7	0
	Bone	101	14	20.5	11	11	16	21	12
	Cartilage	8	1	1	1	1	1	2	1
	Muscle	159	22	21.5	22.5	21	23	17.5	23
	Nerve	2	0.5	0.5	0.5	0.5	0.5	1	0.5
	Tendon	155	21.5	21.5	21.5	24	20	2	26.5
	Other	8	1	1	1	1	1	2	1
	X^2^ (*p*-value)			0.022		0.809		<0.001	
**Cause**									
	Overload	459	64	62	65	60	67	13.5	76
	Degeneration	6	1	1	1	0.5	1	0	1
	Sprain	34	5	6	4	6	4	22	0.5
	Fracture	10	1.5	2.5	1	2	1	4.5	0.5
	Infection	6	1	1.5	0.5	0.5	1	4.5	0
	Lesion	51	7	6	7.5	8.5	6	21	4
	Contusion	38	5	5	5.5	6	4.5	27	0
	Rupture	10	1.5	1	1.5	1.5	1.5	5.5	0.5
	Other	104	14	15	14	15	14	2	17.5
	X^2^ (*p*-value)			0.313		0.570		<0.001	

### Medical Examination

The orthopedic examination related to complaints and symptoms consisted of all aspects to achieve diagnosis and to provide adequate treatment. Besides history and clinical examination of a sports medicine physician, additional data of applied radiological examinations (i.e., sonography, x-ray or MRI) were included into the dataset if applied.

### Data Management

Acute and overuse injuries were specified according to their area, cause and location. Furthermore, specific diagnostics, diagnosis, therapeutic and preventive recommendations and referrals were recorded. By use of preinstalled drop down menus for injury area (head/neck, upper extremity, core/thorax, lower extremity, spine, other), structure (ligament, joint, skin, bone, cartilage, muscle, nerve, tendon, other) and cause (overload, degeneration, sprain, fracture, infection, lesion, contusion, rupture, other) a standardized documentation was guaranteed between different physicians. The term “overload” as a cause of injury describes the reason for the pain symptoms leading to the athletes’ presentation to the physician. “Overload” is referred to both an acute injury (i.e., muscle strain) and pain due to repetitive or chronic overload of a structure (i.e., in tendinopathies) (Kjaer, 2004; Cook and Purdam, 2014). Diagnoses were registered in a free text section (Fuller et al., 2006). In case of an injury involving multiple structures the “main damage” determining the expected recovery time was captured in the system (Fuller et al., 2006).

### Data Analysis

Descriptive analysis was performed by calculation of means and standard deviations (SD). Frequencies were given in absolute (n) and relative numbers (%). Diagnoses sustained were coded with respect to ICD-10 (International Classification of Diseases, 10th version) and analyzed by two independent physicians (Paoin et al., 2018). If necessary for clarification, further chart review of the clinical history was performed. Furthermore, the sample was divided into two age groups (11–14 and 15–17 years of age) in order to identify differences in the injury profile related to age. The cut-off at 14 years of age was set as it has been identified as a critical age in the development of complaints in adolescent elite athletes (Backous et al., 1988; Müller et al., 2017). Additionally, sex differences were analyzed. According to sport-specific loading sport disciplines were allocated into 3 categories [ball and combat sports (BC), endurance sports (E) and explosive strength sports (S); Table 1] and differences considering acute and overuse injuries between the categories were analyzed. Group differences were statistically analyzed by the use of chi-square-tests. Influencing factors (age, sex, and BMI) on area and structure manifestation of injuries were identified using a nominal logistic regression analysis. The overall level of significance was set α = 0.05 (JMP Statistical Software Package 9.0; R, Version 3.2.0).

## Results

A higher proportion of injury-events were reported for females (m/f: 291/427) and for adolescent elite athletes of the older age group (11–14/15–17: 243/475) (Table 2). Injuries were most frequently localized at the lower extremity (47%) followed by the spine (30.5%) and the upper extremity (12.5%). Most often affected structures were joints (34%), muscles (22%) and tendons (21.5%).

### Acute Versus Overuse Injuries

Events of overuse injuries were 3- to 6-fold more frequent than acute injuries concerning different sports categories, with highest proportion for overuse injuries in endurance sports (Table 3). Area, structure and cause were significantly different between acute and overuse injuries (*p* < 0.001; Tables 2, 3). Acute injuries were predominantly located at the lower extremity (73.5%), while overuse injuries were equally observed at the lower extremity (41%) and spine (36.5%) (Table 2). The acute/overuse injury ratio was significantly between sport categories (*p* = 0.009, Table 3), with ball and combat sports showing highest (0.35) and endurance sports (0.17) lowest ratio. For acute injuries, significant differences were observed at the affected area (*p* < 0.001). For overuse injuries, the affected area (*p* < 0.001) and the cause of injury (*p* = 0.037) were significantly different between the sport categories. Ball and combat sports presented more frequently with head/neck injuries, endurance sports with spine injuries and explosive strength sports with lower extremity injuries (Table 3).

**TABLE 3:** Anthropometric data, acute versus overuse injury ratio as well as area, structure, and cause regarding events of acute and overuse injuries separated by sport categories (%; X^2^ *p*-value).

		Ball and combat sports		Endurance sports		Explosive strength sports	
Age (y)		15.8 ± 1.6		15.5 ± 1.5		15.4 ± 1.4	
Height (cm)		173 ± 11		175 ± 10		168 ± 10	
Weight (kg)		64 ± 14		65 ± 12		59 ± 12	
BMI (kg/m^2^)		21.2 ± 2.9		21.3 ± 2.5		20.6 ± 2.5	
Acute/overuse injury ratio*		41/117 (0.35)		46/267 (0.17)		53/194 (0.27)	

		**Acute**	**Overuse**	**Acute**	**Overuse**	**Acute**	**Overuse**
		**(%)**	**(%)**	**(%)**	**(%)**	**(%)**	**(%)**

Area^#^							
	Head/Neck	10	3.5	2	1.5	0	0.5
	Upper Ex	14.5	14.5	6.5	15.5	13	8.5
	Core/thorax	0	4	15.5	12.5	0	4
	Lower Ex	70.5	47	63	31.5	85	50.5
	Spine	2.5	30.5	11	39	2	36.5
	Other	2.5	0.5	2	0	0	0
	X^2^ (*p*-value)	<0.001		<0.001		<0.001	
Structure							
	Ligament	27	1	20	0.5	17	0.5
	Joint	29	27.5	21.5	37.5	28	38
	Skin	2.5	0	11	0	7.5	0
	Bone	19.5	17	26	9.5	19	13
	Cartilage	2.5	1	4.5	1	0	1
	Muscle	10	23	15	21	24.5	27
	Nerve	0	0	2	0	0	0.5
	Tendon	2.5	29	0	30	4	19.5
	Other	7	1.5	0	0.5	0	0.5
	X^2^ (*p*-value)	<0.001		<0.001		<0.001	
Cause°							
	Overload	7	65	11	76.5	21	82
	Degeneration	0	1	0	1	0	1.5
	Sprain	24.5	0	17.5	0.5	24.5	0.5
	Fracture	5	1.5	4.5	0.5	3.5	0.5
	Infection	2.5	0	6.5	0	3,5	0
	Lesion	24.5	8.5	21.5	3	17	2
	Contusion	29	0	28	0	24.5	0
	Rupture	7.5	1	6.5	0	4	0.5
	Other	0	23	4.5	18.5	2	13
	X^2^ (*p*-value)	<0.001		<0.001		<0.001	

*Statistically significant difference for acute/overuse injury ratio between sport categories (*p* = 0.009). ^#^Statistically significant difference for distribution of acute and overuse injuries areas between sport categories (*p* < 0.001). °Statistically significant difference for distribution of overuse injuries causes between sport categories (*p* = 0.037).

### Diagnoses

Overall, the “top 3” diagnoses were segmental hypomobility (“blockage,” 22.5%), tendinopathy (21%) and non-traumatic muscle stiffness (13%; according to ICD-code M62.8; Table 4). For acute injuries, the “top-3” diagnoses were contusion (25%), capsular-ligament lesion (19%) and sprain (17%), while for overuse injuries they were segmental hypomobility (28%) tendinopathy (26%) and non-traumatic muscle stiffness (9%).

**TABLE 4:** “Top 10” diagnoses of the complete dataset (n; %) as well as between subgroups [age groups and sex (n; %)].

Total group			Age group 11–14			Age group 15–17			Males			Females		
	**Count**	**(%)**		**Count**	**(%)**		**Count**	**(%)**		**Count**	**(%)**		**Count**	**(%)**
Segmental hypomobility	160	22.5	Tendinopathy	50	21	Segmental hypomobility	116	24	Tendinopathy	66	23	Segmental hypomobility	103	24
Tendinopathy	150	21	Segmental hypomobility	44	18	Tendinopathy	100	21	Segmental hypomobility	57	20	Tendinopathy	84	20
Muscle stiffness	92	13	Muscle stiffness	31	13	Muscle stiffness	61	13	Muscle stiffness	40	14	Muscle stiffness	53	13
Low back pain	34	5	Osteochondrosis	12	5	Periostitis	24	5	Contusion	16	5	Low back pain	27	6
Contusion	34	5	Low back pain	11	5	Low back pain	23	5	Sprain	14	5	Periostitis	20	5
Periostitis	32	4.5	Contusion	11	5	Contusion	23	5	Cap.-lig. lesion	12	4	Contusion	18	4
Cap.-lig. lesion	26	4	Muscle lesion	10	4	Cap.-lig. lesion	22	5	Periostitis	12	4	Cap.-lig. lesion	14	3
Sprain	24	3.5	Arthralgia	9	4	Sprain	15	3	Low back pain	11	4	Muscle lesion	13	3
Muscle lesion	22	3	Sprain	9	4	Muscle lesion	12	3	Arthralgia	9	3	Arthralgia	10	2
Arthralgia	19	3	Periostitis	8	3	Arthralgia	10	2	Muscle lesion	9	3	Sprain	10	2
Total	593	82.5		195	82		406	86		246	85		352	82

### Influence of Age, Sex, and BMI

**FIGURE 1:**
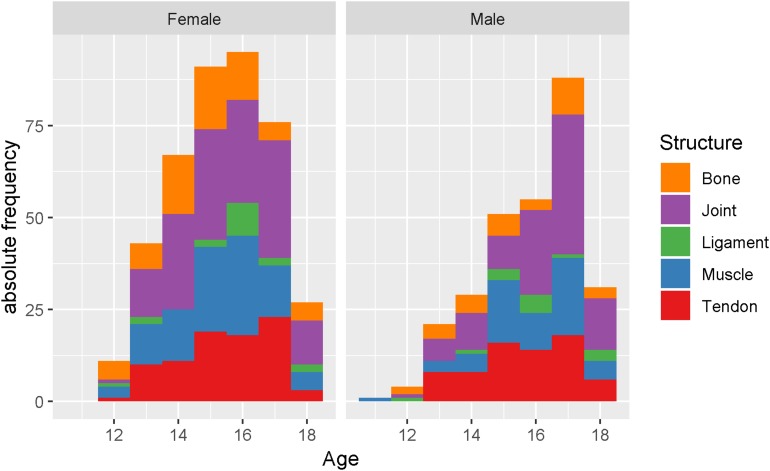
Histogram for “commonly injured structures” by age (years) between sex (left: male; right: female).

The ratio of acute vs. overuse injury-events was higher in males than in females (0.24/0.17, *p* = 0.019), with statistically significance among the older athletes group (11–14 years: 0.27/0.22, *p* = 0.569; 15–17 years: 0.33/0.19, *p* = 0.016). The affected structure was shown to be significantly different between the age groups (*p* = 0.022), with the older age group presenting three times more frequent with ligament pathology events (5.5% vs. 2%) and less frequent with bony problems (11% vs. 20.5%) than their younger counterparts (Table 2 and Figure 1). Logistic regression analysis showed statistically significant influence of factor age on structure bone (*p* = 0.03). Other structures as well as the area of injury-events were not statistically influenced by factor age. The area of injuries was significantly different between sexes (*p* = 0.005). Males presented with fewer spine injury-events (25.5% vs. 34%) but more upper extremity injuries (18% vs. 9%) than females (Table 2). In logistic regression analysis, sex was found to have a significant influence on the areas upper extremity and spine injury-events (*p* < 0.001), while other areas as well as the affected structure was not significantly different between sexes (*p* = 0.809). BMI (corrected by age) resulted in statistically significant influence on both, the structure (ligaments, joints, bones, muscle and tendons; *p* = 0.003) and the area (lower extremity and spine; *p* = 0.040) of injury-events in female athletes.

## Discussion

The study aimed to identify injury profiles in adolescent high performance athletes. Events of overuse injuries of the lower extremity and the spine due to overload were the most frequent cause for presentation at the sports medicine department. Mostly, soft-tissue structures were affected, while segmental hypomobility, tendinopathy and non-traumatic muscle stiffness altogether accounted for more than 50% of the diagnoses. Group differences were found for structure between age groups and for area between sexes. Furthermore, influence of age, sex, and BMI onto injury profiles could be identified.

### Relevance of Overuse Injuries

The results underline the importance to raise the awareness to overuse injuries in adolescent elite athletes (3- to 6-fold higher percentage in different sport categories). This is confirmed by data of Huxley et al. (2014), who have noticed by use of questionnaires that 76% of the injuries were due to overuse in 81 track and field athletes at age 13 to 17 (Huxley et al., 2014). Moreover, Stracciolini et al. (2013, 2014) have reported that 53% of all injuries were overuse injuries in a cohort of 2133 patients aged 5–17 years exercising in “organized physical activity”. Focusing on overuse injuries, the authors have reported significant sex-differences concerning injury location (area) (Stracciolini et al., 2015a). They found that the lower extremity was affected in 61% (f: 67%; m: 53%), upper extremity in 22% (f: 14%; m: 31%) and the spine in 10% (f: 11%; 8%). In contrast, adolescent elite athletes of the present study complained more frequently about symptoms at the spine region and less frequently at the extremities. Most often affected structures were joints, muscles and tendons. In comparison, data of Stracciolini et al. (2013) have suggested the bone to be affected by 61% of cases and soft-tissues only by 33% (Stracciolini et al., 2013). When interpreting this discrepancy between the studies it has to be kept in mind that data of Stracciolini arose from a pediatric medical center of a University hospital. The high proportion of spine overuse injuries in the present cohort might be influenced by the high proportion of athletes from canoe sprint and rowing. Those sports were categorized into endurance sports showing the lowest acute/overuse injury ratio and the highest rates of spine injury events compared to the other sport categories. Moreover, sex distribution regarding different sports in the present data on adolescent elite athletes, with exclusively males performing Handball and Gymnastics, might have an impact on data distribution.

### Influence of Age

Some remarkable age group differences in injury profiles among adolescent elite athletes could also be detected. The older age group presented three times as frequent with injury-events of ligament pathologies and three times as less frequent with bony problems than their younger counterparts. This is reflected by both the three times higher percentage of Osteochondrosis disease events in the younger cohort and the threefold more capsular-ligament lesions in the older cohort. Data of the age group comparison in “organized active” adolescent athletes showed similar results compared to the present study, with the younger age group having more often traumatic and bony injuries compared to the older age group (Stracciolini et al., 2013). In the present study, subanalysis of age group differences in ball and combat sports showed fractures to be solely present in athletes of the young group and ligament lesions exclusively present in the older age group. This might be explained by a significant change in mineralization of the bones already during the initial pubertal phases (Darrow et al., 2009; Kontulainen et al., 2007; Rosendahl and Strouse, 2016; Patel et al., 2017). The increased bony mineralization is believed to lead to a higher bony stiffness and consequently to a higher susceptibility for ligament tears in young athletes (Kontulainen et al., 2007; Darrow et al., 2009).

### Influence of BMI

Influence of BMI on injury profile has been found in adolescent elite athletes as well as in “organized active” adolescent athletes, where BMI was seen as an important factor explaining differences in events of overuse injuries between young males and females (Stracciolini et al., 2015a). BMI as a marker for body composition has already been shown to be a risk factor for various sport injuries of adolescents. A BMI < 17.5 kg/m^2^ in adolescent runners was found to predict low bone mass and goes along with increased risk suffering bony stress lesions (Tenforde et al., 2015). A higher BMI was shown to be associated with higher risk of lower extremity injuries. An increased BMI has led to higher rates of ankle sprain injury in collegiate male athletes and higher rates of ACL injury among “organized active” adolescent athletes (mainly from ball sports and skiing) (Stracciolini et al., 2015b; Hartley et al., 2018). Contrasting this, data of Emery and Tyreman (2009) did not show an increased risk of injury by BMI on multiple regression analysis in young high-school students from 14 different sports (Emery and Tyreman, 2009). For a comprehensive discussion on influence of body composition on injury profiles further evaluation of subcutaneous fat percentage and free muscle mass is required.

### Comparison to Recreationally Active Adolescents

Most of the other available investigations focused on musculoskeletal injuries in recreationally active adolescents. Backous et al. (1988) analyzed injuries during a summer soccer camp including 1139 players aged 6 through 17 years. They found an increase in injury incidence at the age of 14 years. Most frequent were contusions (35%), strains (28%), and sprains (19%) at the lower extremity (70% of all injuries) (Backous et al., 1988). In line with the results of the present study they have reported an increase of injuries with the age of 14 as well as the highest proportion of injuries at the lower extremities. Nevertheless, the comparability of results is limited. The present study assessed all orthopedic injury-events receiving medical attention among adolescent elite athletes’ from 16 different sports, while data during the summer camp were collected solely in recreational soccer players and registered by the coach. Furthermore, acute and overuse injuries were not distinguished in the soccer players (Backous et al., 1988).

Emery and Tyreman (2009) published a cross-sectional study of 1466 students aged 12–15 years reporting sports participation as well as injury rates. They found highest participation in game sports (basketball, soccer, volleyball). Lower extremity injuries and concussions accounted for over 60% and 15% of all injuries with the highest sport-specific injury rates in ice hockey, rugby, basketball, soccer, and American football (Emery and Tyreman, 2009). With the data of the present study it is not possible to calculate injury rates per sport due to the fact that absolute participation rate is unknown. However, the results showed comparable injury frequencies of the lower extremities in ball and combat sports. Fridman et al. (2013) evaluated approximately 57000 recreational and sports-related injuries out of 13 different types of sports among children and youth athletes aged 5–19 years. Highest injury rates with 57% were found at age group 10–14 years. About 30% of all injury types were fractures. In the interpretation of those data it has to be considered that they arose from presentations to Canadian emergency departments (Fridman et al., 2013). Contrasting this, data in the present study was evaluated from the sports medical departments in the federal state responsible for athletes care. Not surprisingly, adolescent elite athletes did not show as often fractures, concussions, ACL- or meniscus lesions and spondylolysis but higher frequencies of overuse injuries (Backous et al., 1988; Steffen et al., 2008; Darrow et al., 2009).

### Top Diagnoses

It has recently been shown that prevalence and incidence of back pain as well as of lower extremity tendinopathies are underreported in young adolescent elite athletes (Cassel et al., 2015, 2018; Simpson et al., 2016; Müller et al., 2017). Due to the long-standing and the potentially chronifying character of the diseases early diagnostics and clear treatment strategies are mandatory, especially in adolescent elite athletes (Cook et al., 2016; Simpson et al., 2016; Hartvigsen et al., 2018). Currently, active treatment of tendinopathies (i.e., eccentric exercises) is evident solely for adult populations (Young et al., 2005; Alfredson and Cook, 2007). Eccentric training was shown to improve pain status in tendinopathic patients (Alfredson and Pietila, 1998; Kingma et al., 2007; van der Plas et al., 2012) and leads to an increase in tendon stiffness in healthy subjects (Malliaras et al., 2013; Bohm et al., 2015). However, its impact on tendinopathic tendon mechanical properties is not clear, yet. Tendon stiffness among adolescents (aged 9–14 years) was shown to be lower compared to healthy adults (O’Brien et al., 2010; Intziegianni et al., 2016). The imbalance of high muscle strength and relatively compliant tendon structure leading to high stress on the tendon material is discussed as a risk factor for the development of tendinopathy (Mersmann et al., 2014, 2016). This stress-strain-relationship of tendons might play a crucial role for the higher susceptibility of tendinopathies of young adolescent elite athletes in the present cohort (Mersmann et al., 2014, 2016). As a consequence, development of prevention strategies should also be focused on active loading (i.e., eccentric exercises) of the lower extremity tendons in order to strengthen the tendon structure (Mersmann et al., 2014, 2016, 2017). High frequency of overuse injuries already at young age supports the need for an implementation of prevention programs early in adolescent athlete’s career.

### Limitations

When interpreting the results of the presentations with acute symptoms it has to be considered that the investigated elite athletes were screened for eligibility in pre-participation examinations prior to entrance to an elite school of sport (Mayer et al., 2012). Therein, entrance of athletes at high risk to sustain or to progress diseases has been refused (Mayer et al., 2012). Noteworthy, the included adolescent elite athletes originated from 16 different sports. Since the injury profiles of various sports can be considerably different, generalizability of the data is limited. By distinguishing between sport categories this limiting factor is partly addressed. Unfortunately, the exact number of sport school students in each sport is unknown. Therefore, calculation of (normalized) injury rates to the number of participants in each sport was not possible. Likewise, it cannot be excluded, that the findings are skewed due to a higher participation rate of athletes among specific sport.

Statistically, it has to be mentioned, that the Chi-square test does not evaluate the magnitude of differences between categories. It was therefore not possible to determine how practically important the observed differences were. Furthermore, it has to be taken into account, that a large sample size increases the likelihood of obtaining a statistically significant result that may not necessarily be practically important (Hopkins et al., 2009).

Furthermore, it has to be kept in mind, that the primary sports medicine departments included in the study did not have emergency admission, including 24 h emergency service. This might have led to an underestimation of total traumatic injury occurrence. Moreover, several physicians were included into the data acquisition, which could have led to an observer bias. A standardized digital documentation platform has been used to minimize this documentation bias between different physicians.

## Conclusion

Soft-tissue overuse injuries are the most common reasons in adolescent elite athletes leading to a medical presentation. Especially older female adolescent elite athletes seem to be at risk suffering from orthopedic injury-events. Mostly, the lower extremity and the spine are affected, while sex and age characteristics on affected area and structure must be considered. Segmental hypomobility, tendinopathy and non-traumatic muscle stiffness represent more than 50% of the documented diagnoses. Prevention strategies should therefore be implemented already in early adolescence and should especially focus on the prevention of overload injury-events of joints, tendons and muscles at the lower extremity and the spine. Furthermore, prevention strategies should be developed sports-specific and with respect to age, sex, BMI as well as injury entity (acute and overuse).

## Data Availability

The datasets generated for this study are available on request to the corresponding author.

## Ethics Statement

This study was carried out in accordance with the recommendations of the “Ethics committee of the human faculty of the University of Potsdam” with written informed consent from all subjects. All subjects gave written informed consent in accordance with the Declaration of Helsinki. The protocol was approved by the “name of committee.” “The clinical review committee has no objections.”

## Author Contributions

MC and FM conceived or designed the study and acquired the data. MC, JM, OM, JR, and FM interpreted the data. MC and MS drafted the manuscript. MC, JM, OM, JR, and FM revised the manuscript. All authors analyzed the data, approved the final version of the manuscript to be published and agreed to be accountable for all aspects of the work.

## Conflict of Interest Statement

The authors declare that the research was conducted in the absence of any commercial or financial relationships that could be construed as a potential conflict of interest.
